# Crosstalk between Wnt/β-catenin signaling pathway and DNA damage response in cancer: a new direction for overcoming therapy resistance

**DOI:** 10.3389/fphar.2023.1230822

**Published:** 2023-08-02

**Authors:** Xixia Zhang, Xiaofeng Yu

**Affiliations:** Department of Otolaryngology Head and Neck Surgery, Shengjing Hospital of China Medical University, Shenyang, China

**Keywords:** Wnt, DNA damage response, cancer, therapy, drug resistance

## Abstract

Wnt signaling plays an important role in regulating the biological behavior of cancers, and many drugs targeting this signaling have been developed. Recently, a series of research have revealed that Wnt signaling could regulate DNA damage response (DDR) which is crucial for maintaining the genomic integrity in cells and closely related to cancer genome instability. Many drugs have been developed to target DNA damage response in cancers. Notably, different components of the Wnt and DDR pathways are involved in crosstalk, forming a complex regulatory network and providing new opportunities for cancer therapy. Here, we provide a brief overview of Wnt signaling and DDR in the field of cancer research and review the interactions between these two pathways. Finally, we also discuss the possibility of therapeutic agents targeting Wnt and DDR as potential cancer treatment strategies.

## 1 Introduction

The Wnt signaling pathway is a complex protein regulatory network cascade, which participates in various biological processes, including embryonic development, tissue development and regeneration, and cancer development and progression. The abnormal activation of Wnt signaling will result in many pathological processes, such as cancer, inflammatory and immune diseases, and metabolic diseases (Clevers and Nusse, 2012; Nusse and Clevers, 2017).

DNA damage response (DDR) is a hierarchical signaling pathway that functions to maintain the genome integrity and stability and is coordinated by a variety of proteins. Numerous studies have demonstrated that DDR deficiency is closely associated with several diseases (Jackson and Bartek, 2009; Ciccia and Elledge, 2010; Lee and Paull, 2021; Stoof et al., 2021; Ye et al., 2021), among which cancer has become a focus of research. DDR has been extensively involved in regulation of cell senescence, carcinogenesis, and cancer progression as well as influencing the efficacy of cancer radiotherapy and chemotherapy. As the majority of anti-tumor therapies primarily target the genomic DNA of cancer cells, enhancement of DDR is closely related to their therapeutic efficacy (Lord and Ashworth, 2012; Su et al., 2023; Traphagen et al., 2023).

Previous studies have reported that Wnt signaling can affect DDR by regulating a variety of factors (Lento et al., 2014; Tao et al., 2015; Liang et al., 2018). In this review, we first introduce the Wnt/β-catenin and DDR signaling pathways and focus on the role of signaling in the regulation of DDR. Wnt/β-catenin pathway and DDR pathway members are potential therapeutic targets for many types of cancer (Lord and Ashworth, 2012; Krishnamurthy and Kurzrock, 2018; Pilié et al., 2019; Wang et al., 2021a). Could this possibly shed new light on clinical decision-making? We describe the molecular mechanisms and associations of the Wnt and DDR pathways with the biological processes involved in cancer and the treatment of cancer and their potential impact on the development of new treatment regimens.

## 2 Wnt signaling pathway

**FIGURE 1:**
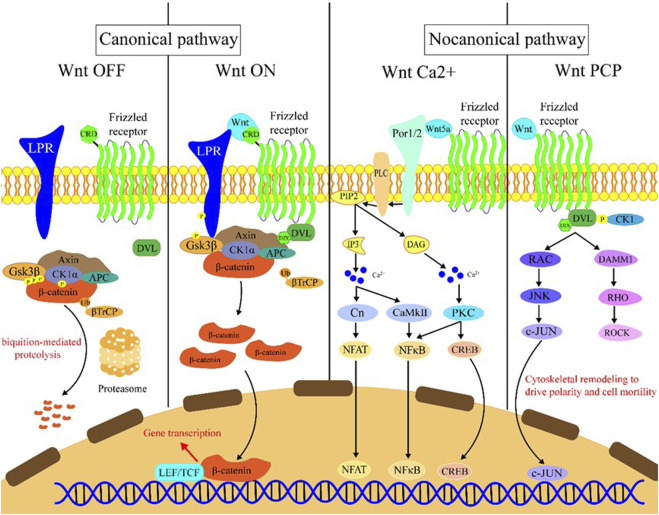
Introduction of the Wnt signaling pathway. Wnt OFF: In the absence of Wnt ligand, β-catenin binds to the destruction complex which comprises AXIN, CK1α, GSK-3β, and APC. Subsequently, a series of phosphorylation events occur to create a docking site on β-catenin for β-TrCP, and then β-catenin is ubiquitinated by β-TrCP and subjected to ubiquitin-proteasomal degradation. Wnt ON: In the presence of Wnt ligands, Frizzled receptors and the co-receptors LRP5/6 multimerize at the cell surface. This leads to recruitment of the cytoplasmic protein Dvl to the cell membrane by interacting with cytoplasmic domains of Frizzled receptors. The Frizzled-bound Dvl recruits the destruction complex through Dvl and axin, GSK-3β in the destruction complex initiates phosphorylation of the LRP5/6 and subsequent phosphorylation by CK1s, and more destruction complexes are recruited to the cell membrane that further phosphorylate LRP5/6 as a positive feedback loop. Wnt Ca^2+^: Wnt/Frizzled ligand receptor interaction with the participating co-receptor ROR1/2 leads to the activation of PLC and then hydrolyzes PIP2 into products IP3 and DAG. IP3 causes the release of Ca^2+^ from the endoplasmic reticulum, and two kinases CaMKII and Cn are activated, which in turn activate NFAT and NF-κB. DAG is activated by released calcium from the endoplasmic reticulum. Subsequently, PKC is activated which then activates NF-κB and CREB. These factors translocate to the nucleus where downstream gene expression is regulated. Wnt PCP: In the planar cell polarity pathway, after binding to Frizzled receptors and recruiting Dvl, which forms a complex with DAAM1, Wnt then activates the small GTPase Rho, which in turn activates ROCK. Alternatively, Dvl could also form a complex with RAC to activate JNK and then increase JNK-dependent c-JUN transcription activity.

The Wnt signaling can be activated through the binding of Wnt family member ligand to the membrane receptors like Frizzled (FZD) family receptors, low-density lipoprotein receptor-related protein 5/6 (LRP5/6), and receptor tyrosine kinase-like orphan receptors (ROR1/2). Wnt signaling activation leads to transcriptional activation of multiple downstream effectors (Clevers and Nusse, 2012), and extracellular signals are transmitted through this pathway into the cell via activation of the intracellular segments of receptors on the cell surface, which in turn activates β-catenin-dependent or -independent Wnt signaling cascades. There are several separate Wnt pathways which can engage in crosstalk with one another; these have been summarized as canonical (β-catenin-dependent) and non-canonical signaling (Wnt/Ca^2+^ (calcium) pathway and Wnt/PCP (planar cell polarity) pathway) pathways (Figure 1). Generally, the canonical Wnt signaling pathway mainly participates in regulating progenitor cell self-renewal, proliferation, or differentiation, while the non-canonical signaling pathway is primarily responsible for the maintenance of cell stemness, cell motility, or antagonism of the canonical pathway (Fodde and Brabletz, 2007; Clevers and Nusse, 2012; Nusse and Clevers, 2017).

### 2.1 The canonical Wnt/β-catenin pathway

The key members of the canonical Wnt signaling pathway include the Wnt family proteins, FZD/LRP6, disheveled protein (Dvl), β-catenin, T lymphocytokine/lymphoenhancer factor (TCF), human lymphoid enhancer factor (LEF), and the β-catenin destruction complex which comprises adenomatous polyposis coli (APC), axin, glycogen synthase kinase-3β (GSK-3β), and casein kinase 1α (CK1α). In the absence of a Wnt signal, β-catenin binds to the destruction complex, resulting in its ubiquitination via proteasomal degradation mediated by β-TrCP. In the presence of Wnt signal, Wnt binds to the FZD and LRP5/6 receptors to activate Dvl protein and inhibit the destruction complex, resulting in the accumulation of free unphosphorylated β-catenin in the cytoplasm, which is then transported to the nucleus where it can combine with TCF/LEF to induce transcription of downstream Wnt signaling genes (Clevers and Nusse, 2012; Nusse and Clevers, 2017).

### 2.2 The non-canonical Wnt pathway

#### 2.2.1 The Wnt/Ca^2+^ pathway

The Wnt proteins could bind to the FZD receptors on the cell transmembrane and participate in several cellular processes that are involved in stimulating the isotrimer G protein to further activate PLC. PLC activation could result in elevated release of intracellular Ca^2+^, reduced cyclic guanosine (cGMP) levels, and activation of Ca^2+^/calmodulin-dependent protein kinase-II (CaMKII) and PKC; these processes can stimulate NFAT and other transcription factors such as CREB1 (De, 2011).

#### 2.2.2 Wnt/PCP signaling

In Wnt/PCP signaling (Wang et al., 2021a), PCP mainly regulates cell movement direction and cell morphology. Wnt ligands bind to the ROR–FZD receptor complex to recruit and activate Dvl which binds to the small GTPase Rho. Then, the small GTPase Rac1 and Rho together trigger ROCK (Rho kinase) and JNK, thereby contributing to the regulation of cytoskeletal and transcriptional responses.

## 3 DDR regulators and signaling pathways

Under physiological conditions, DNA damage can be caused by various environmental stimuli and intracellular stress. After suffering DNA damage, cells can have three possible fates: recovering to the normal physiological state, initiation of apoptosis, or survival with damage. Through various regulatory pathways, cells can efficiently recognize DNA damage lesions, activate the DDR, and initiate the corresponding repair mechanism. The DDR involves a series of complex and elaborate regulatory events, including the identification of lesions, activation of checkpoints, remodeling of chromatin, DNA damage repair, cell cycle arrest, and apoptosis.

The DNA repair pathways vary in response to different types of DNA damage, which can be classified as follows: base excision repair (BER), nucleotide excision repair (NER), mismatch repair (MMR), homologous recombination (HR; where another nucleotide chain is required as a repair template), and non-homologous end-joining (NHEJ, where the broken ends are directly connected to the DNA strand). HR and NHEJ are the main repair ways for double-strand breaks (DSBs). HR always occurs in G2 and S phases, during which sister chromatids are present and can provide a template strand, while NHEJ is cell cycle phase-independent. For eukaryotes, NHEJ repairs most DSBs, and HR is preferentially used when the lesion occurs at DNA replication forks.

**FIGURE 2:**
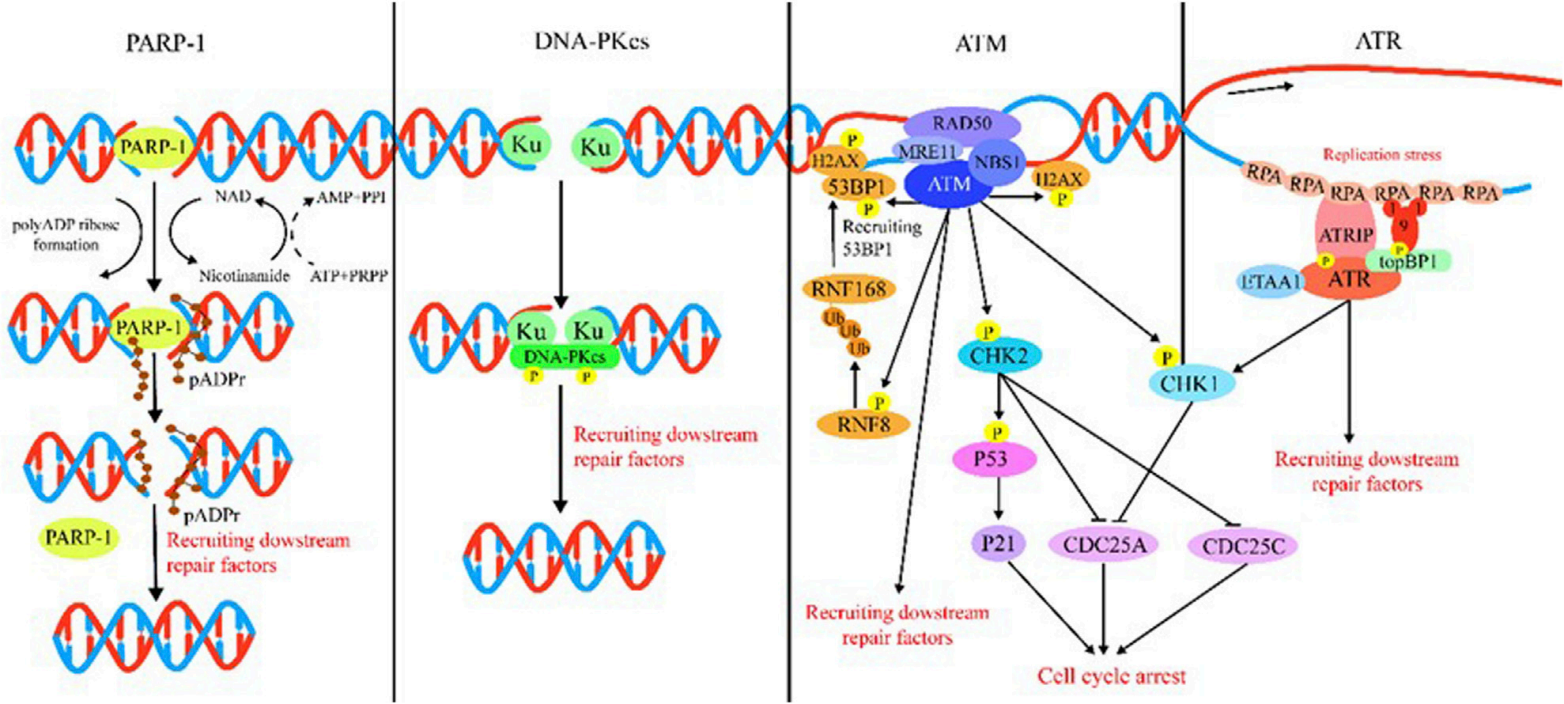
Introduction of DDR. PARP-1: PARP-1 detects and binds to damaged DNA and subsequently synthesizes poly (ADP) ribose (pADPr) on acceptor proteins. The high-density negative charge of pADPr gradually accumulates, leading to PARP-1 release from DNA simultaneously with the recruitment of DNA repair protein complex. Lastly, after the DNA break is repaired, the repair complex is dissociated from DNA and poly (ADP-ribose) glycohydrolase (PARG), and ADP-ribose hydrolase 3 (ARH3) hydrolyzes pADPr into ADP-ribose molecules and free pADPr. DNA-PKcs: DSBs are rapidly bound by the Ku heterodimer (Ku70 and Ku80) and loads onto DSB ends. Within seconds, Ku loads and activates DNA-PKcs to initiate NHEJ. Additional NHEJ core factors are subsequently recruited for the ends to be closely aligned and ligated. ATM: The MRN complex recruits ATM to DNA lesions and stimulates ATM kinase activity in response to DSBs. ATM phosphorylates histone H2AX and MDC1 in response to DSBs and lays the foundation for the chromatin-based signaling cascade involving phosphorylation and ubiquitination, which are mediated by RNF8 and RNF168 that results in ubiquitination of H2A to promote recruitment of 53BP1, which recruits its effectors to repair lesion. In addition, ATM phosphorylation activates Chk1/2, which in turn influences downstream factor cell to arrest the cell cycle. ATR: After suffering various forms of damaged DNA or helicase-polymerase uncoupling at stalled replication forks, RPA coats the ssDNA protecting it from degradation. ATR is recruited to RPA-ssDNA by its partner protein ATRIP and activated by TopBP1 or ETAA1; TopBP1 plays a role in ATR activation, which requires interaction with the RAD9–HUS1–RAD1 (9-1-1) clamp complex. ETAA1 is recruited to RPA-ssDNA via direct binding to RPA. In the downstream, ATR signaling activates the CHK1 kinase. CHK1 activation causes CDC25A degradation and slowing of cell cycle progression to arrest the cell cycle.

DDR is majorly regulated by two protein families: the phosphatidylinositol 3-kinase-like protein kinase (PIKK) family (including ATM, ATR, and DNA-PK) and the 16-member poly (ADP) ribose polymerase (PARP) family. Generally speaking, ATM, ATR, DNA-PK, and PARP mainly function as sensors for DNA damage. ATM is recruited by the MRN (MRE11/RAD50/NBS1) complex following recognition of DSBs (Rupnik et al., 2010; Blackford and Jackson, 2017), and ATM is the kinase typically responsible for modulation of cellular responses to DSBs, which comprise DNA repair, checkpoint activation, apoptosis, senescence, and alterations in the chromatin structure (Shiloh and Ziv, 2013; Blackford and Jackson, 2017). ATR is recruited by 9-1-1 (RAD9–RAD1–HUS1) complex to extend tracts of ssDNA (Blackford and Jackson, 2017). ATR is the DNA replication stress response kinase which could phosphorylate numerous substrates under the stimulation of genotoxic stresses. Following detection of DSBs, DNA-PK is recruited by Ku to initiate NHEJ (Graham et al., 2016). Among PARP family proteins, the structure and function of PARP-1 have been well elucidated by researchers. PARP plays a multifaceted role in cellular response to DNA damage. For example, PARP acts as a sensor in DDR that catalyzes the addition of poly (ADP-ribose) chains to histones and other nuclear proteins to recruit DDR factors to chromatin at breakpoints (Amé et al., 2004). PARP could also cause chromatin remodeling and recruit DNA-repair-associated proteins (Haince et al., 2008). In Figure 2, we summarize the DDR processes involving these different sensors, namely, ATM, ATR, DNA-PK, and PARP.

The downstream effects of these repair processes involve activation of various substrates to elicit appropriate responses involved in cell fate determination. These downstream effectors include H2AX, CHK1, CHK2, and p53, among others. H2AX is phosphorylated to form γH2AX, which is one of the earliest events in DDR. γH2AX not only generates a signaling cascade to amplify the signal of DDR and recruits the DDR proteins but also regulates chromatin relaxation (Rogakou et al., 1998). CHK1 and CHK2, coordinating DDR by arresting the cell cycle (Abraham, 2001), can phosphorylate p53 at Ser20 (Bode and Dong, 2004). P53 plays an important role in activating repair proteins to promote DNA repair, arresting the cell cycle in G1/S to provide ample time to repair damage, and initiating apoptosis to prevent abnormal genetic information from dividing and growing if the damage proves to be irreparable (Shiloh and Ziv, 2013; Li et al., 2016). Several characteristics of p53 are particularly notable. In the normal physiological state, p53 is presented at low levels in cells because of proteasome-mediated degradation, which is closely related to complex formation with various types of E3 ubiquitin ligases, the most important of which is Mdm2 (Brooks and Gu, 2006). HIPK2, a p53 Ser46 kinase, is retained in an inactive status because of the targeted proteolysis by E3 ubiquitin ligases in unstressed cells, making it an important regulator of stress signaling and DDR (Matt and Hofmann, 2016). When DNA damage is encountered, ATM and ATR kinases could phosphorylate Siah1, resulting in the dissociation of the HIPK2–Siah1 complex and increased stability of the HIPK2 protein (Winter et al., 2008).

## 4 Wnt and its crosstalk with DDR

**FIGURE 3:**
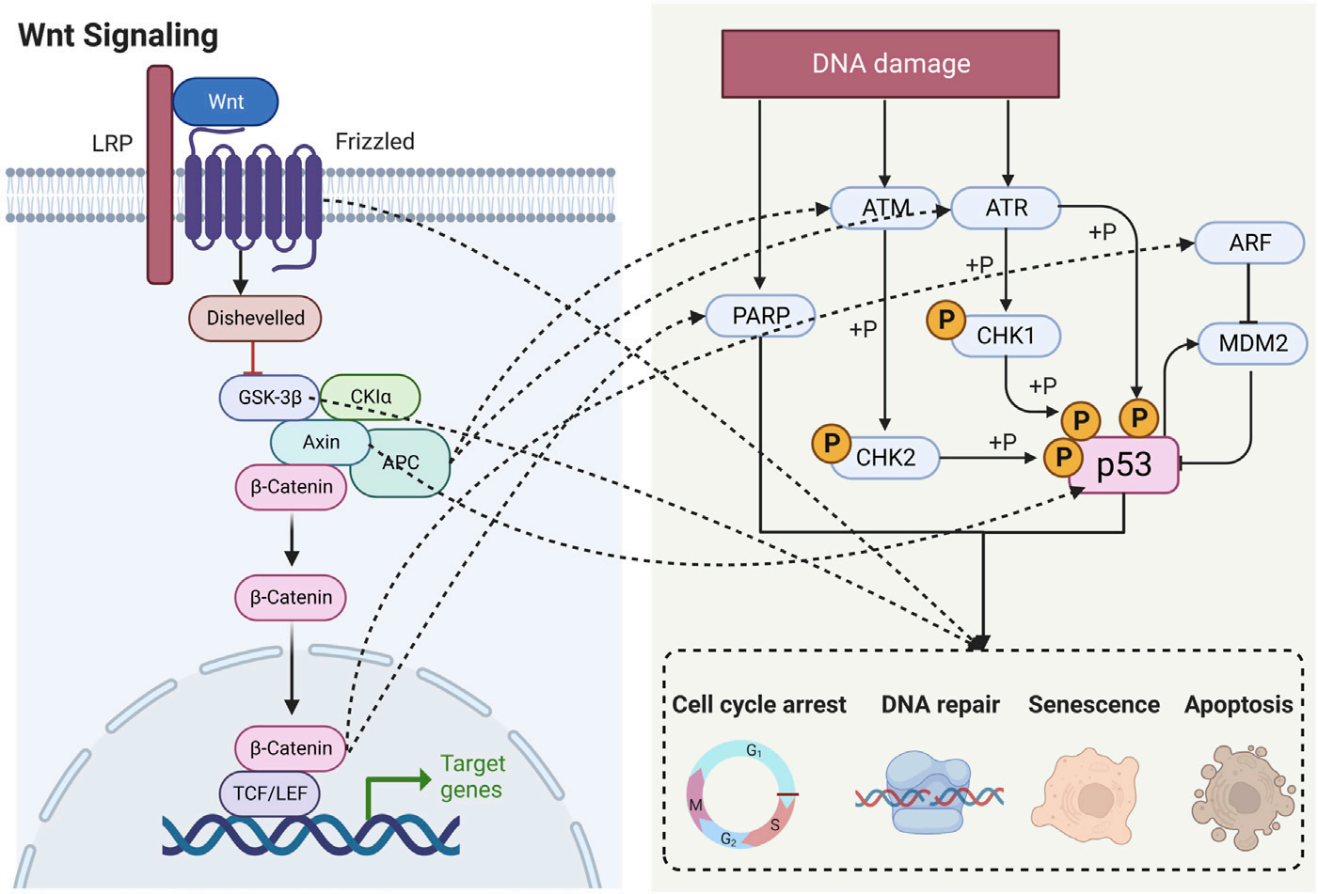
Schematic diagram showing that Wnt signaling is highly intertwined with DDR. LRP, lipoprotein receptor-related protein; APC, adenomatous polyposis coli; GSK-3β, glycogen synthase kinase-3β; CK1α, casein kinase 1α; TCF, T lymphocytokine/lymphoenhancer factor; LEF, human lymphoid enhancer factor; PARP, poly (ADP) ribose polymerase; ATM, ataxia telangiectasia mutated proteins; ATR, ataxia telangiectasia and Rad3-related protein; CHK, checkpoint kinase; ARF, ADP-ribosylation factor; MDM2, murine double minute 2.

Numerous studies have shown that Wnt signaling is highly intertwined with DDR (Zhang et al., 2011; Karimaian et al., 2017; Zhao et al., 2018b). In this section, we elaborate on these specific connections by outlining the roles of key factors (Figure 3).

### 4.1 The role of β-catenin in DDR

β-catenin is implicated in the regulation of DDR (Xu et al., 2008; Zhang et al., 2011; Priolli et al., 2013; Tavana et al., 2013; Chandra et al., 2015; Serebryannyy et al., 2017). Activation of downstream target genes of β-catenin such as cyclin D1 and c-myc proto-oncogenes leads to cell proliferation, differentiation, and maturation (Tulac et al., 2003). Notably, there is a growing list of Wnt/β-catenin target genes which have been found in various species and have been cataloged at: http://www.stanford.edu/group/nusselab/cgi-bin/wnt/target_genes. Based on these data, we have summarized the target genes closely related to DDR and briefly describe their roles in DDR.

### 4.2 The role of APC in DDR

APC is a key effector of the canonical Wnt signaling pathway involved in downregulating the β-catenin level. Many previous studies have shown that the loss of APC function can cause chromosomal instability (Fodde et al., 2001; Kaplan et al., 2001; Bienz, 2002). Clarke et al. found that APC loss leads to DNA damage and genomic instability in the live cell in a process closely associated with p53 (Méniel et al., 2015). In addition, there have been reports that APC is involved in the BER (Jaiswal and Narayan, 2008; Jaiswal and Narayan, 2011). Moreover, APC loss decreases cancer cell sensitivity to chemotherapy by reducing phosphorylation of ATM/Chk1/Chk2, which in turn influences DDR (Stefanski et al., 2019). APC loss can also increase STAT3 activation, leading to chemotherapy resistance (VanKlompenberg et al., 2017). STAT3 has been shown to take part in DNA repair, as low STAT3 levels can reduce ATM and ATR signaling through MDC1 (Barry et al., 2010).

### 4.3 Regulation of Axin on DDR

Axin, a key scaffolding protein, is responsible for the formation of the β-catenin destruction complex. Stability of axin protein is regulated by the ubiquitin-proteasome system, and enhancing axin stabilization is a common method of inhibiting Wnt/β-catenin signaling. In DDR, axin interacts with PML to regulate p53-dependent apoptosis in response to DNA damage (Li et al., 2011). Axin is involved in regulating phosphorylation of p53 Ser46 by forming a p53–axin–HIPK2 complex (Rui et al., 2004). Additionally, Lin et al. found that Tip60 interacts with axin in an ATM/ATR/CHK1-dependent way and abrogates Pirh2–axin binding, forming an axin–Tip60–HIPK2–p53 complex and leading to p53 phosphorylation in response to genotoxic stress during radiochemotherapy (Li et al., 2009).

### 4.4 Regulation of FZD on DDR

FZD is the seven-pass transmembrane receptor of Wnt. FZD5 is a member of the FZD family. Zhao et al. revealed that FZD5 has been involved in triple-negative breast cancer (TNBC) cell G1/S transition and DNA damage repair partially dependent on Wnt7B (Sun et al., 2020). Moreover, FZD5 modulates the downstream effecter FOXM1 in a Wnt/β-catenin-dependent manner to regulate the cell cycle, DNA replication, and DNA damage repair (Sun et al., 2020). In addition, a study on ovarian cancer found that another FZD family member, FZD7, can protect cells from chemotherapy-induced oxidative stress through the FZD7–β-catenin–Tp63–GPX4 pathway (Wang et al., 2021b). Similarly, another study showed that FZD10 can regulate Wnt signaling in BRCA-mutated epithelial ovarian cancers, ultimately contributing to increased HR activity (Fukumoto et al., 2019).

### 4.5 The effects of GSK-3β on DDR

GSK-3β is involved in multiple intracellular signaling pathways and is a component of the β-catenin destruction complex in Wnt signaling, which determines β-catenin stabilization. In DDR, GSK-3β can phosphorylate several DNA repair factors and affect their interaction with chromatin. For instance, through phosphorylating cyclin D1, CDC25A, and CRY2, GSK-3β can tune DNA repair and cell cycle (Diehl et al., 1998; Harada et al., 2005; Kang et al., 2008). Furthermore, GSK-3β can also phosphorylate B-Myb, leading to the dissociation of B-Myb from the MRN-mediated response to DNA damage (Henrich et al., 2017). Translin-associated protein X (TRAX) is a DNA/RNA-binding protein that participates in various functions, including DNA repair and physical interaction with GSK-3β and other DNA repair factors, to further influence NHEJ-mediated repair (Weng et al., 2018). In the cytoplasm, GSK-3β could phosphorylate targets to trigger proteasomal degradation and promote activation of NF-κB activity to evade apoptosis (Lin et al., 2020). On radiation exposure, GSK-3β translocates from the cytoplasm to the nucleus and phosphorylates 53BP1, an important regulator for DNA repair (Yang et al., 2018). There is strong evidence that GSK-3β plays an important role in DDR; however, whether those functions of GSK-3β depend on Wnt/β-catenin signaling requires further investigation.

### 4.6 The role of p53 in DDR

In DDR, p53 can be activated in various ways. For example, ATM and ATR can phosphorylate p53 on Ser15 which is thought to inhibit interaction of p53 with the ubiquitin ligase, MDM2, leading to its dissociation from MDM2, thereby contributing to p53 stabilization (Shiloh and Ziv, 2013). Likewise, the p53 co-factor, hnRNPK, can be phosphorylated by ATM and protect p53 from MDM2-mediated degradation (Moumen et al., 2013). In the Wnt pathway, Wnt/β-catenin can promote the expression of ARF, which binds to MDM2 leading to its inactivation (Kubbutat et al., 1997). In contrast, the activation of p53 could trigger the Siah/SIP/Skp1/Ebi pathway for β-catenin ubiquitination degradation and then reduce activity of TCF/LEF (Matsuzawa and Reed, 2001). Accordingly, p53 is a key factor in the crosstalk between the Wnt/β-catenin signaling pathway and DDR.

### 4.7 Regulation of PARP on DDR

In general, PARP, a DNA damage sensor, is activated by recognizing damaged DNA fragments. DNA damage would induce auto-polyADP-ribosylation of the PARP-1 protein to inhibit the functional interaction of PARP-1 with TCF-4 and participate in the transcriptional regulation of TCF-4/β-catenin complex target genes, such as *cyclin D1* and *c-myc* (Idogawa et al., 2005). In addition, Ku70 is an inhibitor of the β-catenin/TCF-4 transcriptional complex, and PARP-1 could compete with Ku70 for binding to TCF-4, thereby modifying transcriptional activity of TCF-4 (Idogawa et al., 2007). Moreover, there is considerable evidence that Wnt/β-catenin signaling activation can promote resistance to PARP inhibitors (Fukumoto et al., 2019; Yamamoto et al., 2019; Zhu et al., 2021). Therefore, PARP may provide a novel linkage between Wnt/β-catenin signaling and DDR.

In summary, there are many connections between Wnt and DDR (Table 1); however, whether these factors regulate DDR directly via Wnt signaling or independent of the Wnt signaling pathway warrants further investigation.

**TABLE 1:** Function of Wnt target genes in DDR.

Gene	Function in DDR
c-myc	Regulates the cell cycle, telomere homeostasis, and c-myc target genes are involved in DDR, DNA synthesis, and apoptosis
n-myc	Regulates DDR
Cyclin D1	Regulates cell cycle and DNA repair
uPAR	Activates DNA repair signaling pathway
MMP-7	DNA repair
Endothelin-1	Enhances DNA repair
Jagged1	DNA repair
iNOS	Product NO causes DNA damage and inhibits DNA repair proteins
Telomerase	Sustains DNA damage signals in senescent cells, regulates apoptosis, and protects the cell against DNA damage
Sox9	Increases cell survival and regulates the cell cycle
Sox17	Downregulation of DDR genes
Runx2	Regulates the cell cycle, DNA repair, and apoptosis
SALL4	Activates the critical ATM-dependent cellular responses and chromatin remodeling
Osteoprotegerin	Reduces UV-induced apoptosis
CCN1 (Cyr61)	Regulates the cell cycle
Sox2	Regulates the cell cycle
PTTG (securin)	Regulates cell cycle, activates DDR, and modulates DDR gene expression
Nanog	Deregulates DDR and chromatin remodeling
OCT-4	Regulates the homologous recombination, regulates the cell cycle, and modulation of p53
Fibronectin	Regulates the cell cycle and priming and potentiation of DDR
Wnt3a	Regulates the cell cycle
Connexin43	Regulates the cell cycle
RARγ	Involved in DNA damage-induced necroptosis and extrinsic apoptosis
MITF	DNA repair, activates DDR signaling cascade, and regulates the cell cycle
Stra6	Apoptosis
Autotaxin	Involved in DDR and DNA repair
WISP	Apoptosis
COX-2	Prevents DNA damage
Nkx2.2	Regulates ATM activity
WISP-1	Regulates p53-mediated apoptosis
Periostin	Periostin-deficient embryos could be linked to improve DNA damage repair and regulates the cell cycle
β-TrCP	Regulates the cell cycle and DNA repair
CDC25A	Regulates the cell cycle
P16	Regulates the cell cycle and induces DDR

## 5 Potential treatment strategies

At present, initiation and increase of DNA damage in cancer cell is a commonly exploited strategy for treating cancer and plays an effective therapeutic role; however, some cancer cells can develop resistance to DNA damage drugs by activating DDR. Increased DNA repair leads to resistance of cancer cells to targeted local (radiotherapy) or systemic (chemotherapy) treatments. Numerous kinds of drugs used for cancer chemotherapy, such as Adriamycin, etoposide, bleomycin, and cisplatin, are characterized as intense inducers of DNA damage and could effectively activate the intracellular DDR (Jackson and Bartek, 2009). All parts of DDR require proper coordination to maintain genome stability, and they can all influence the effects of cancer treatment. Therefore, DDR components can serve as radiation and chemical sensitization targets in cancer treatment. Knowledge of the DDR defects that are present in cancer can also allow for the selection of optimal treatments that can efficiently kill the cancer cells (Goldstein and Kastan, 2015). Currently, many drugs targeting the DDR have been developed to treat tumors, which are shown in Schedule 1. A summary of clinical trials on Wnt/β-catenin-targeted agents is presented in Table 2.

**TABLE 2:** Drug target and clinical trials on Wnt/β-catenin-targeted agents in cancer.

Target	Mechanism of action	Compound name	Cancer type	Trial identifier	Reference
PORCN	Porcupine inhibitor	LGK974 (Wnt974)	Pancreatic cancer, BRAF mutant colorectal cancer, melanoma, triple-negative breast cancer, head and neck squamous cell cancer, cervical squamous cell cancer, esophageal squamous cell cancer, and lung squamous cell cancer	NCT01351103	Rodon et al. (2021)
			Metastatic colorectal cancer	NCT02278133	Tabernero et al. (2023)
		ETC-159 (ETC-1922159)	Solid tumors	NCT02521844	Katoh (2018)
		RXC004	Colorectal cancer	NCT04907539	
			Advanced solid tumors	NCT04907851	Phillips et al. (2022)
			Cancer and solid tumors	NCT03447470	
		CGX1321	Colorectal adenocarcinoma, gastric adenocarcinoma, pancreatic adenocarcinoma, bile duct carcinoma, hepatocellular carcinoma, esophageal carcinoma, and gastrointestinal cancer	NCT03507998	Li et al., 2019; Goldsberry et al., 2020; Shah et al., 2021
Wnt	Preventing Wnt binding to FZD	Ipafricept (OMP-54F28)	Hepatocellular cancer and liver cancer	NCT02069145	Jimeno et al., 2017
			Ovarian cancer	NCT02092363	Moore et al. (2019)
			Pancreatic cancer and stage IV pancreatic cancer	NCT02050178	
			Solid tumors	NCT01608867	
	Wnt5a agonist	Foxy-5	Metastatic breast cancer, colorectal cancer, and prostate cancer	NCT02020291 and NCT02655952	Säfholm et al., 2008; Canesin et al., 2017; Osman et al., 2019
			Colon cancer	NCT03883802	
FZD	Inhibits Wnt signaling by binding FZD 1, 2, 5, 7, 8	Vantictumab (OMP-18R5)	Solid tumors	NCT01345201 and NCT01957007	
			Pancreatic cancer and stage IV pancreatic cancer	NCT02005315	Davis et al., 2020; Diamond et al., 2020
			Metastatic breast cancer	NCT01973309	
	FZD10 antagonist	OTSA101-DTPA-90Y	Synovial sarcoma	NCT01469975	Goswami and Patel (2021)
	FZD8 decoy receptor	Ipafricept (OMP-54F28)	Solid tumors	NCT01608867	
			Hepatocellular cancer and liver cancer	NCT02069145	Jimeno et al., 2017; Moore et al., 2019; Dotan et al., 2020
			Ovarian cancer	NCT02092363	
			Pancreatic cancer	NCT02050178	
ROR1	Anti-ROR1 antibody	Cirmtuzumab (UC-961)	Chronic lymphocytic leukemia	NCT02860676	
				NCT02222688 and NCT03088878	
			Metastatic castration-resistant prostate cancer	NCT05156905	Choi et al., 2015; Choi et al., 2018
			Chronic lymphocytic leukemia	NCT04501939, NCT02860676, and NCT02222688	
			Breast neoplasms	NCT02776917	
			B-cell chronic lymphocytic leukemia, small lymphocytic lymphoma, and mantle cell lymphoma	NCT03088878	
		NBE-002	Advanced solid tumor, advanced cancer, and triple-negative breast cancer	NCT04441099	
	Overexpressing ROR1	Zilovertamab vedotin (VLS-101 and MK-2140)	Hematologic malignancies	NCT03833180	
			Solid tumors	NCT04504916	Kipps, 2021; Vaisitti et al., 2021
			Relapsed or refractory diffuse large B-cell lymphoma	NCT05139017	
		NVG-111 (RP2D)	Chronic lymphocytic leukemia and mantle cell lymphoma	NCT04763083	
	Targeting of genetically engineered autologous T-lymphocytes to ROR1^+^ tumors	Anti-ROR1 CAR	ROR1^+^ tumors	NCT02706392	
ROR2	Targeting of genetically engineered autologous T-lymphocytes to ROR2^+^ tumors	CCT301CAR (CCT301-38 and CCT301-59)	Renal cell carcinoma	NCT03393936	
			Solid tumor, soft tissue sarcoma, gastric cancer, pancreatic cancer, and bladder cancer	NCT03960060	
	Conditionally active biologic anti-ROR2 antibody drug conjugate	Ozuriftamab vedotin (CAB-ROR2-ADC and BA3021)	Non-small cell lung cancer, triple-negative breast cancer, melanoma, and head and neck cancer	NCT03504488	
			Recurrent or metastatic squamous cell cancer of the head and neck	NCT05271604	
			Ovarian cancer	NCT04918186	
			Non-small cell lung cancer	NCT04681131	
CBP	CBP/β-catenin antagonist	PRI-724	Colorectal cancer	NCT02413853 and NCT04351009	
			Advanced or metastatic pancreatic cancer	NCT01764477	
			Advanced myeloid malignancies	NCT01606579	
			Advanced solid tumors	NCT01302405	
β-catenin	Degrading β-catenin	CWP232291	Acute myeloid leukemia and chronic myelomonocytic leukemia	NCT01398462	Pak et al., 2019; Lee et al., 2020; Wang et al., 2022
			Acute myeloid leukemia	NCT03055286	
			Multiple myeloma	NCT02426723	

Theoretically, targeting both DDR and Wnt signaling could serve as potential treatment strategies for cancer. Another common denominator of them is that DDR and Wnt signaling have been associated with therapeutic resistance (Martin-Orozco et al., 2019). Alterations in Wnt signaling are also closely associated with maintenance of cancer stem cell (CSCs) proliferation, as hyperactivation of the Wnt signaling pathway is critical in supporting cancer cell survival in the context of treatment with anti-cancer drugs and ultimately leads to cancer progression. Therefore, the design and development of appropriate anti-cancer strategies based on Wnt targets may have important therapeutic value in treating cancer cells, thereby enhancing the therapeutic efficacy of these strategies (Mukherjee and Panda, 2020; Zhang and Wang, 2020). The formation of drug resistance could be attributed to some different mechanisms such as the acquisition of quiescence, the interaction with the microenvironment, drug efflux capacity, increased resistance to apoptosis, and increased DNA repair. For example, FZD5 enhances DNA damage repair and chemoresistance (Sun et al., 2020). GSK-3β promotes resistance of cancer cells to DNA damage chemotherapeutic agents and radiation through regulating DNA repair and stemness of cancer cells (Lin et al., 2020). YAP/TAZ is a Wnt regulator that can be activated to protect cancer cells from DNA damage (de Lau et al., 2014). Furthermore, the role of Wnt signaling in promoting DNA damage repair and inhibiting apoptosis contributes to cancer resistance to radiation (Jun et al., 2016; Zhao et al., 2018a).

Most current anti-cancer chemotherapy approaches function by killing highly proliferating cells, which in many cancers are mostly non-CSCs. However, small CSC populations present in cancer have a higher repair mechanism and are also highly resistant to chemotherapy. In addition, radiotherapy and chemotherapy may trigger a series of cellular stress response mechanisms that enhance stem cell properties of non-CSCs, thereby improving their adaptation and survival. Therefore, compared with previous therapies, new combination therapies, which can kill both CSCs and non-CSCs while preventing the transition from non-CSCs to CSCs, require consideration. It would be possible to develop a therapeutic strategy which combines DDR-targeting drugs with Wnt-targeting drugs to increase sensitivity of cytotoxic drugs while contributing anti-CSC effects. Importantly, the connection points between the DDR and Wnt warrant further investigation, as drugs targeting these points are likely to inhibit both DDR and CSCs simultaneously.

## 6 Discussion and conclusion

In the context of cancer, increased Wnt signaling is beneficial and reflected in enhancement of DDR, endowing cancer cells with increased ability to repair themselves during radiation or chemotherapy. The association between Wnt signaling and DDR in the post-carcinogenesis stage have been well studied, but the relationship between these two pathways during the process of carcinogenesis requires further investigation. There is a strong correlation between carcinogenesis and inadequate repair of DNA damage; however, whether Wnt signaling is involved in this process remains unknown. Future studies are required to investigate whether Wnt signaling can influence carcinogenesis via regulation of DDR.

Although Wnt signaling and the DDR are closely related, the effects generated by different specific points of crosstalk are unknown. Identification of an appropriate regulator as a therapeutic target may be beneficial to cancer treatment. Compared with single-drug chemotherapy or radiotherapy, drug combination may achieve double efficacy with half the input. From another perspective, a triple therapy including DDR-targeting drugs and Wnt-targeting drugs has the potential to enhance the efficacy of radiotherapy and chemotherapy. Although our theory may be considered oversimplified, identification of crosstalk between the Wnt signaling pathway and DNA damage recognition has potential to provide novel insights into cancer therapy.
